# De novo synthesis of synthetic biology ecosystem in Slovakia: Challenges and opportunities

**DOI:** 10.1016/j.biotno.2022.06.001

**Published:** 2022-07-05

**Authors:** Miroslav Gasparek, Jakub Hantabal

**Affiliations:** a Department of Engineering Science, University of Oxford, Parks Road, Oxford, OX1 3PJ, United Kingdom; b Institute of Biological, Environmental and Rural Sciences, Aberystwyth University, Aberystwyth, SY233FL, United Kingdom

## Abstract

Synthetic biology is an engineering discipline that applies engineering principles to rationally design novel biological systems. It has the potential to contribute to solving major global challenges in a multitude of areas, from healthcare to sustainability. While the engineering biology landscape is robust and well-established in certain countries, the ecosystem and infrastructure for genetic engineering in other countries, including Slovakia, are underdeveloped. Consequently, such countries are missing the major economic and social benefits that the practical applications of the rational design of biological systems may provide. In this work, we briefly assess the status of the synthetic biology landscape in Slovakia in different areas, including research efforts, industrial participation, governmental policy, and the educational landscape. We describe the major challenges that the Slovak synthetic biology sector faces and propose a strategy that academics, policymakers, and industry could take to activate the proliferation of the Slovak synthetic biology ecosystem.

## 1 Introduction

Life in every organism composed of cells is governed by a complex interplay of signalling pathways, metabolism and regulatory components. Synthetic biology aims to understand and harness components of these pathways at every level.^1^ This engineering discipline also focuses on the development of frameworks for the rational design of novel biological systems through the application of fundamental engineering principles, such as abstraction, characterization, standardization, and modularization.^2^^,^^3^ Following the key initial breakthroughs including the genetic toggle switch^4^ and repressilator^5^ at the beginning of the 21st century, rational design of biological systems benefited from advances in technical capabilities of DNA and RNA sequencing and synthesis, as well as the decreasing costs of these foundational tools of synthetic biology.^6^ At the same time, the availability of mathematical and computational design tools for engineered biological systems has improved significantly.^7^ Consequently, engineering biology has transformed from an intellectually stimulating intersection of engineering, systems theory and molecular biology that once drew the attention of a small number of engineers and biologists, to one of the most important engineering disciplines with major economic potential and societal impacts.^8^

Combining improved understanding and rational design approach should lead to numerous practical applications in healthcare,^9^ sustainability,^10^ industry,^11^, ^12^, ^13^ and many other areas of modern society. Indeed, synthetic biologists are developing novel therapeutic^14^ and diagnostic modalities,^15^ produce novel drug delivery systems,^16^ discover new ways of decreasing the costs of bioproduction of industrial compounds^17^ and support sustainability efforts.^10^ Overall, synthetic biology is a major contributor to the global bioeconomy, which might have a direct economic impact of up to $4 trillion a year in the next two decades.^18^

Many countries have recognized the potential of engineering biology education, research and industry. These countries including the United Kingdom, the United States and Singapore have devoted significant resources not only to the planning of their national synthetic biology strategies,^19^, ^20^, ^21^ but also to focusing on synthetic biology curriculum^22^, ^23^, ^24^ investing in engineering biology research,^25^^,^^26^ support of entrepreneurial efforts in rational biosystems design, and assistance in establishing of national synthetic biology ecosystems.^27^^,^^28^

Unfortunately, the development of the engineering biology landscape in Slovakia has been trailing behind the leading countries not only the countries that are leading global development but also behind the neighbouring Central and Eastern European countries. Slovakia is one of the global leaders in the automotive industry^29^ and has a strong tradition in heavy industry. A major emphasis has been given on the development in Information Technology (IT) and digitalisation sectors over the last two decades, which gave rise to IT companies such as ESET, Sygic, and Pixel Federation.^30^ However, the advanced synthetic biology industry is largely absent in the local economy and research in engineering biology is not well-established when compared to the neighbouring countries. Sadly, the lack of focused research effort spans across multiple areas of synthetic biology, from computational modelling and design to biosecurity.^31^^,^^32^

The underdevelopment of the synthetic biology ecosystem in Slovakia represents a major missed economic opportunity and, given modern biosecurity challenges,^33^ it might also pose a threat to the national security. This perspective aims to describe the synthetic biology landscape in Slovakia. We provide a summary of the current synthetic biology opportunities in Slovakia. Subsequently, we pinpoint the key factors contributing to Slovakia's sub-optimal performance in the field. We will also map the current key players, institutions and organisations contributing to the development of synthetic biology in Slovakia. Additionally, we discuss structural challenges that hinder the development of the Slovak synthetic biology sector and outline the specific steps that should be taken to improve the status of the synthetic biology landscape in Slovakia. We believe that these challenges are not necessarily unique to Slovakia; rather, our work aspires to serve as a guide for researchers and policymakers in other countries in which synthetic biology has not been an area of interest.

## 2 The current Slovak synthetic biology ecosystem

The current Slovak synthetic biology ecosystem has an upper bound given by the Slovak biotechnology industry, which is itself not receiving sufficient attention from individual stakeholders. The key players who can impact the synthetic biology industry in Slovakia include academia, government, industry, entrepreneurs, educational institutions, and media. Here, we describe the state and impact of each of these players in reference to the Slovak synthetic biology landscape.

### 2.1 Research

The major public higher education institutions that are completing research related to synthetic biology are the Biomedical centre of the Slovak Academy of Sciences (BMC) and the Institute of Biotechnology (IoB) at the Slovak University of Technology in Bratislava. The BMC is one of the many institutes integrated within the structure of the Slovak Academy of Sciences and it focuses on research bridging the gap between the laboratory and the clinic. One of the key focus areas of BMC is the development of cancer therapeutics, where methods of synthetic biology could be applied to a larger extent.

However, synthetic biology has not been routinely employed in the research at the BMC in the past. 2020 Annual report by BMC revealed that only two research projects employed synthetic biology, namely “*Preparation of new antibiotics and antitumour agents by manipulations of secondary metabolite genes and synthetic biology methods*” and “*Activation of VGF/BDNF/TrkB pathways by synthetic mRNA encapsulated in polyplex nanoparticles: effects on nerve excitability, neuroplasticity and animal behaviour*”.^34^ The further heading of these projects, as well as their potential to succeed is unclear, given that both projects are underfunded and do not leverage the opportunities of international collaboration.^34^ Nevertheless, BMC and associated Institute of Virology in particular have demonstrated a keen interest in applications of the tools of synthetic biology in some of its key investigations, particularly the investigation of the impact of Carbonic Anhydrase IX on tumour microenvironment,^35^^,^^36^ the flagship research of this institution conducted in collaboration with numerous internationally recognized research groups.

Apart from the tumour microenvironment, the focus on industrial bioproduction by researchers at the IoB of the Slovak University of Technology in Bratislava has been another major area of focus of synthetic biology efforts in Slovakia. The relevant projects include the conversion of low-cost materials into high-value organic molecules, such as polyunsaturated fatty acids in yeast *Yarrowia lipolytica*.^37^^,^^38^ This area of research is of high interest because of its potential industrial value.^39^ Furthermore, this research is conducted in collaboration with internationally recognized research groups, including researchers at Masaryk University in Brno and Imperial College London in the UK. Overall, we believe that industrial bioproduction of high-value compounds in engineered organisms represents a promising industrial direction. Slovakia possesses the foundations to employ synthetic biology in bioproduction - one of the epicentres of the Slovak biotech industry in Slovakia is the town of Slovenska Lupca, where companies including Biotika (specialising in the production of drugs 2 and vaccines) and Evonik Fermas (specialising in upscale and production of biological substances) are based. However, we have not identified any applications of advanced synthetic biology techniques by these companies.

Research of engineering biology in Slovakia is governed by the European Union (EU) regulations, including the Cartagena protocol for Biosafety.^40^ While strict, these regulations do not currently restrict the synthetic biology research in Slovakia. This is partially due to the early stage this research is in, as ambitious, upscalable research projects are not yet in development.

### 2.2 Governmental initiatives

To the knowledge of the authors, no national synthetic biology strategy focused on the development of synthetic biology exists on the national level. In addition to that, the industry reports by Slovak Investment and Trade Development Agency (SARIO), which is responsible for supporting industrial ecosystems in Slovakia do not discuss synthetic biology at all.^41^ A positive example of interaction between public institutions and the synthetic biology research community is that the Ministry of Environment of the Slovak Republic employs a scientific advisor for synthetic biology, who has collaborated and co-authored with some of the leaders in the field.^42^

There is currently limited collaboration with the European Union and the United Nations in the research of applications of synthetic biology in developing alternative energy sources, however, information available on this project is limited to a brief note of its existence in the final report of the Operational Programme of Integrated infrastructure.^43^

### 2.3 Startups and emerging companies

While multiple successful software companies have been built and headquartered in Slovakia, including ESET, Exponea (acquired by Bloomreach) and Slido (acquired by Cisco), there is only a limited number of true biotechnology startups. The most eminent example is MultiplexDX, which attracted international attention during the COVID-19 pandemic thanks to its development and distribution of rapid and accurate Polymerase Chain Reaction and Loop-mediated isothermal amplification diagnostic tests in a time of national urgency.^44^^,^^45^ While there are other biotechnology companies, we have not been able to identify any startups specifically naming synthetic biology as its key technology.

In addition to that, the translatability and upscale of organisms generated through rational biological systems design is hindered by the strict European regulations for genetically-modified organisms (GMOs).^46^ Therefore, European Union and, indeed, Slovakia would benefit from a comprehensive roadmap of implementation of engineering biology to the biotechnological industry, akin to the strategies developed in the UK.^47^^,^^48^

### 2.4 Education

The Comenius University and the Slovak University of Technology are two leading institutions in education in life sciences. A dedicated synthetic biology course is not offered, however, disciplines partially related to engineering biology are taught, including molecular biology, genetics, chemical engineering and biotechnology. We believe that this is a consequence of the lack of collaboration between the life science and engineering departments, as engineering departments generally specialise in mechanical engineering due to related to the automotive industry, the industrial powerhouse of Slovakia. In addition to that, the high school, as well as the higher education in Slovakia suffers from its narrow focus on individual subjects, which is antithetic to the interdisciplinary nature of synthetic biology. Furthermore, popular initiatives such as International Genetically Engineered Machine (iGEM) competition and Biomolecular Design Competition (BIOMOD competition) have never been run in Slovakia. Improving communication and coordination between the biological and engineering university departments would provide students with opportunities to extensively explore synthetic biology throughout their studies.

### 2.5 Public perception of synthetic biology

In the countries with more advanced synthetic biology ecosystem, the discipline has found its way into mainstream media and is more frequently mentioned with positive connotations. Media are working towards improving its public perception and providing a complete picture of what can be achieved in the field.^49^ In contrast, the Slovak public remains uneducated about the discipline, as the major media attempt to cater for the broad 3 public with limited understanding of science, who are easily impressed by interesting and futuristic-sounding headlines.^50^ Such narrative results in an oversimplification of the concepts, therefore, most of the Slovak population either does not know about synthetic biology or perceive it in a negative way, as an enigmatic, futuristic threat. Media coverage often provides a narrative concentrating on the emphasis on the potential risks of genetically engineered cellular elements, similar to the narrative against genetically-modified crops that has been widely promoted for years.^51^ The effort to articulate not only the potential risks of synthetic biology but also its advantages is largely absent. Sadly, this is a worldwide issue; indeed, synthetic biology is often portrayed as risky or dangerous.^52^ Similarly to the public, certain politicians and persons with some scientific knowledge with impact on policymakers may also be influenced by the negative perception of synthetic biology, thus becoming reluctant to advance engineering biology research in Slovakia. Balanced discussions with experts in the field with emphasis on the positive impact of the field for society are needed to shift the perception of genetic engineering from the negativistic, anti-GMO narrative to recognition of the advances potentially made when synthetic biology experiments are pursued.

## 3 Activating the synthetic biology ecosystem in Slovakia

**Fig. 1:**
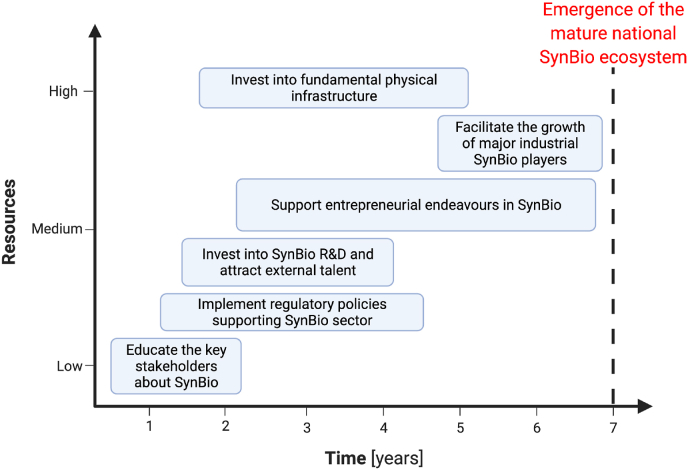
The roadmap for the development of the Slovak national Synthetic Biology (SynBio) ecosystem. The chart depicts the time and relative amount of resources (e.g. human, financial) that are required to successfully build a mature synthetic biology landscape. The first step is to educate the key stakeholders, including public officials, investors, and academics, about the opportunities and challenges of synthetic biology. Subsequently, the appropriate regulatory policies should be implemented at a national level. Investments into synthetic biology R & D and engagement of synthetic biology experts from abroad should be executed afterwards. Investments into fundamental physical infrastructure (i.e. lab spaces and synthetic biology equipment) should be enacted in parallel with supporting the entrepreneurial endeavors in synthetic biology. Finally, as the last step towards the maturity of the developing synthetic biology ecosystem, the public administration should facilitate the emergence of the major Slovak or foreign industrial players that would operate in synthetic biology sector, bring sustained investments and drive the rise of synthetic biology sector as a stable part of the national economy.

The building of the synthetic biology sector in Slovakia faces multiple challenges. However, there are specific actions that the community of enlightened researchers, governmental officials, entrepreneurs and private investors can take to significantly accelerate the proliferation of synthetic biology in Slovakia. The development of a healthy national synthetic biology sector requires an orchestrated effort on multiple fronts over the period of several years (Fig. 1).

### 3.1 Educate the key stakeholders about synthetic biology

A good biotech ecosystem can function because of mutualism between researchers, industry, private investors, and government. This is currently not conceivable in Slovakia because, apart from scientists, other stakeholders are largely unaware of the opportunities provided by synthetic biology. For instance, one of the key focus areas of the Slovak government is the support of Hydrogen-enabled industry.^53^ Synthetic biology provides engineering solutions that could enable further development of the Hydrogen industry in Slovakia.^54^^,^^55^ However, the government is still largely unaware of the opportunities in this space.^56^

Therefore, further education of every stakeholder in the ecosystem is needed. Conferences dedicated to synthetic biology or the inclusion of synthetic biology topics in other relevant conferences would provide opportunities for the scientific community to educate the investors, allowing them to realize the business potential of synthetic biology.

The personal development of scientists starts during the completion of their undergraduate degrees. It is therefore important that students are presented with a wide selection of opportunities and extracurricular activities, allowing them to gain deep insight into topics of their interest, leading to the student's specialisation in the future. It is important that synthetic biology is a part of such extracurricular options. In fact, the infrastructure to achieve so is already present in Slovakia - the Slovak Academy of Sciences hosts talks for high school and university students on their ongoing research and traditional laboratory methods and their applications. The inclusion of synthetic biology into such talks is easily achievable by inviting experts in the field from abroad. Additionally, meetings of these global synthetic biology leaders with the representatives of the Slovak government would help the officials to obtain insight into the appropriate governance, allowing for improved decisions to be made on the policy level, for example on funding synthetic biology research and investing in building physical infrastructure for biotechnology research.

### 3.2 Attract international synthetic biology experts

Senior scientists should help train the early-career researchers and serve as their mentors. As the availability of active senior synthetic biology researchers in Slovakia is extremely limited, it is apparent that such mentors need to be brought from abroad. As a European Union (EU) member state, Slovakia has the potential to leverage EU sources to support cutting-edge research. However, apart from funding, intimate scientific collaborations should be established in order for Slovakia to retain its top synthetic biology talent. While the Slovak Academy of Sciences is the leading scientific institution in Slovakia, its complex organisational structure and administrative hurdles result in difficulties in implementing requisite changes. Private enterprises, however, could perhaps provide such a dynamic environment. Currently, Slovakia is experiencing a change in the approaches to research and medicine, driven by private investments in healthcare. Such undertakings could contribute to making Slovakia attractive to the established synthetic biologist, who would facilitate the exchange of knowledge between the established researchers and early-career researchers. Furthermore, participation in student-led competitions, such as iGEM could be a viable route to engaging young bioengineers.

### 3.3 Support startups and industrial initiatives

The most developed national synthetic biology ecosystems (and biotech ecosystems in general) appreciate the contribution of startups and the business sector towards the advancement of research. Additionally, in the biotech hubs such as London, San Francisco, and Boston, there is the appropriate infrastructure supporting networking between entrepreneurs, researchers, and venture capital investors. These, for example, include laboratory spaces for lease, which, in contrast, are not readily available in Slovakia. This is a major hurdle for early-stage startups, which may experience difficulties with finding appropriate wet lab places to work on their products in early stages while facing the lack of venture capital that would support such development. Importantly, the absence of senior talent and scientists with deep technical knowledge coupled with business acumen is a major hurdle that early-stage synthetic biology startups in Slovakia have to face.

Therefore, building appropriate lab facilities in Slovakia could represent a massively positive step toward the support of innovative companies. Realization of projects related to the development of such infrastructure could be beneficial for both startups and investors, as such spaces could prove to be profitable in the long term. Additionally, Slovakia could potentially leverage EU development funds for the development of this support structure.

## 4 Conclusion

The ability of humankind to engineer novel biological systems has grown exponentially since the beginning of the millennium. There are still major technical challenges that have to be overcome in order for humans to fully enjoy the fruits of synthetic biology and, simultaneously, perhaps even more difficult ethical and biopolicy questions that have to be considered. However, the unequal distribution of the capacity to engineer new living systems across the globe is one of the major questions that have to be addressed. Countries such as Slovakia, where the synthetic biology sector is significantly underdeveloped may suffer from economic and social drawbacks, such as increased vulnerability to climate change and decreased access to novel medicines. Therefore, supporting the development of synthetic biology capabilities should be among the key priorities of all relevant stakeholders.

In this work, we described the state of the synthetic biology sector in the Slovak Republic. Synthetic biology has, unfortunately, received relatively lower attention than the automotive industry or IT. However, the global pandemic of COVID-19 should serve as a wake-up call not only for policymakers, but also for all stakeholders in the ecosystem. The first step that has to be made is educating key players about synthetic biology, its potential to deliver social and economic benefits, as well as its possible threats. Subsequently, the effort to retain top Slovak synthetic biology talent in the country and prevent it from fleeing abroad must be made, especially through engaging with the top, internationally recognized experts in the field. Furthermore, the opportunity to learn from the experts from other countries, which are ahead of Slovakia in terms of maturity of synthetic biology sectors, should be exploited. Finally, the investments into infrastructure, the support of biotechnology entrepreneurship and collaboration with industry must be encouraged in order to help transform Slovakia into the synthetic biology leader, at least at a local level.

## Declaration of interests

The authors declare that they have no known competing financial interests or personal relationships that could have appeared to influence the work reported in this paper.
